# Antioxidative and Metabolic Responses in Canola: Strategies with Wood Distillate and Sugarcane Bagasse Ash for Improved Growth under Abiotic Stress

**DOI:** 10.3390/plants13152152

**Published:** 2024-08-03

**Authors:** Emad M. Hafez, Yan Gao, Khadiga Alharbi, Wei Chen, Nevien Elhawat, Tarek Alshaal, Hany S. Osman

**Affiliations:** 1 Department of Agronomy, Faculty of Agriculture, Kafrelsheikh University, Kafr El-Sheikh 33516, Egypt; emadhafez2014@gmail.com; 2 Institute of Agricultural Resources and Environment, Jiangsu Academy of Agricultural Sciences, Nanjing 210014, China; wchen@jaas.ac.cn; 3 Key Laboratory of Agro-Environment Downstream of Yangze Plain, Ministry of Agriculture and Rural Affairs of the People’s Republic of China, Nanjing 210014, China; 4 Department of Biology, College of Science, Princess Nourah bint Abdulrahman University, P.O. Box 84428, Riyadh 11671, Saudi Arabia; kralharbi@pnu.edu.sa; 5 Department of Applied Plant Biology, Faculty of Agricultural and Food Sciences and Environmental Management, University of Debrecen, Böszörményi str. 138, 4032 Debrecen, Hungary; alshaal.tarek@agr.unideb.hu; 6 Faculty of Agriculture (Girls), Al-Azhar University, Cairo 11884, Egypt; 7 Soil and Water Department, Faculty of Agriculture, Kafrelsheikh University, Kafr El-Sheikh 33516, Egypt; 8 Department of Agricultural Botany, Faculty of Agriculture, Ain Shams University, Hadayek Shubra, Cairo 11241, Egypt; hany_osman1@agr.asu.edu.eg

**Keywords:** antioxidants, canola, drought, deficit irrigation, salt-affected soil, sugarcane bagasse ash, wood distillate, physiological responses

## Abstract

In the context of increasing agricultural challenges posed by soil salinity and drought stress, the main importance of the present study was to evaluate some novel treatments for improving canola productivity and resilience by applying wood distillate (WD) in combination with bagasse ash (SBA). A two-year field experiment using a split plot design was conducted and evaluated several physiological and biochemical parameters under different irrigation regimes conducted at 80% and 50% field capacity. While there were considerable moderation effects of SBA and WD on soil salinity, expressed as exchangeable sodium percentage (ESP), under both well-irrigated and drought conditions, more importantly, the ESP was reduced to 31% under drought stress with combined WD and SBA applications over any single factor. WD and SBA treatments of canola leaves showed reduced Na content with increased K levels, and the plants maintained physiological attributes—chlorophyll content, stomatal conductance, and relative water content—to the level of controls of well-irrigation. Besides, they significantly alleviated oxidative stress by decreasing the hydrogen peroxide (H_2_O_2_), malondialdehyde (MDA), and electrolyte leakage (EL) levels and increasing the activities of antioxidant enzymes like superoxide dismutase (SOD) and ascorbate peroxidase (APX). Nonenzymatic antioxidants such as total soluble sugars (TSS), total soluble proteins (TSP), total phenolic content (TPC), and total flavonoid content (TFC) were significantly increased under stress conditions with a special accent on combined treatment, whereas the levels of proline and GB that increased in alignment with drought reduced under the combined application. Various growth parameters of plants like plant height, number of branches, and siliques per plant were significantly improved with WD and SBA under drought stress. Principal component analysis (PCA) and Pearson correlation further confirmed the relationships among these parameters and thus underpinned that WD and SBA can evoke a synergistic effect to enhance growth promotion and stress tolerance in canola. This, therefore, infers that the combined application of WD and SBA can be key, offering very high potential as viable options to better canola productivity under adverse environmental conditions.

## 1. Introduction

Canola (*Brassica napus* L.) has been cultivated for a long time in order to obtain edible oil as the canola crop is considered the second largest crop after the cultivation of soybean for the production of oil rapeseeds [1,2]. According to the FAO [3], it was found that the annual production capacity of canola oil has reached 26 million tons. The relative importance of the canola crop is due to its richness in polyunsaturated fatty acids, which is considered a healthy food oil. In addition to the by-product resulting from squeezing the seeds, which is used as animal feed because it contains a high percentage of protein (around 50%) [4], canola oil also has other uses such as lubrication and paint, and recently, it has been used in the bioenergy industry [5]. However, with the huge annual increase in population (around 2 billion people by the year 2050), the increase in crops due to the cultivated area is still insufficient to meet consumption [6] as a result of the impact of climate changes that increase saline soils and exposure to drought in some regions around the world, especially in Egypt [7]. These abiotic stresses increase the negative impact on productivity as well as the quality of the oil, causing a decrease in its value as well as its profitability [8,9].

The exposure of plants to drought and low soil fertility due to the effect of high salinity are the most widespread factors resulting from climate change that negatively affect the growth and development of plants [10,11]. These abiotic stresses cause a decrease in soil quality and reduce the availability of water necessary for the growth of field crops [12]. All of this is due to high temperatures and a decrease in rainfall resulting from climate change [13,14]. Abiotic stresses cause a decrease in soil permeability [15], a lack of nutrients in the soil due to increasing Na^+^ ions and decreasing K^+^ ions [16] as well as metabolic activities and difficulty in absorbing nutrients from the soil to the roots [17] and the translocation and distribution of the minerals within the plant. This causes complete disruption to the physiological and biochemical characteristics of the leaf [18], inhibiting cell expansion and division and reducing the concentration of chlorophyll and the efficiency of photosynthesis resulting from reactive oxygen species (ROS) [19], which increases oxidative stress and cell damage [20]. Hence, it is crucial to develop effective techniques to ameliorate degraded saline soils and expose plants to drought to maintain sustainable crop growth and improve rapeseed crop production [21,22].

Using different soil applications as one of the most useful and safest strategies can improve soil fertility and help retain the soil water content around the rhizosphere zone [23]. It can reduce the harmful effects of salinity and drought together, which reflects positively on the growth and development of plants and improves the quality of the oil and crop production [24].

Wood distillate (WD) or pyroligneous acid is a transparent reddish-brown liquid that comes from a green biomass material that is made by condensing the smoke produced during biochar production as a by-product of pyrolyzed wood that is heated under high temperature and hypoxia [25]. It primarily consists of acetic acid, butyric acid, catechol, phenol, and organic acids [26]. Wood distillate has a positive impact on stimulating plant development as a plant growth promoter or biostimulant [27]. Furthermore, it can decrease the harmful effects of abiotic stressors [28]. Reports have illustrated that WD enhances seedlings and hastens the root growth and other plant parts aboveground [2,26] due to it containing organic components of guaiacol, pyrogallol, and syringol [29]. These organic compounds have the ability to increase antioxidant activities, which help eliminate reactive oxygen species (ROS) and alleviate the damage impact of abiotic stress [30]. Moreover, applying WD could improve the nutrient availability and soil microbial structure, which enhances stress resistance; such impacts can eventually increase the productivity and quality [2]. Preceding reports illustrated that foliar spraying with WD could stimulate the seedlings and growth, augment dry weight, and improve the resilience to water stress due to its positive effect on improving antioxidant enzyme activity and soluble protein and nutrient uptake [31].

Sugarcane bagasse, as a fibrous plant residue, is one of the major biomass resources of the Egyptian sugar industry, with a current yearly production of about 2.7 million tons [32]. This feedstock is incompletely burned, leading to aerial releases, carbon loss, and air contamination [33]. Bagasse, as a by-product, is considered as a renewable energy source as well as a soil amendment in the form of composted ash to enhance soil health and quality. It also has a wide range of environmental advantages (as an eco-friendly product that is cost-effective), where it contains up to 40% of organic matter (i.e., charcoal), and the rest corresponds to inorganic materials (mostly silica) [34]. It has been shown that the addition of sugarcane bagasse ash (SBA) improved the soil enzyme activities along with plant development in soils co-affected with drought and salinity [35]. SBA contains some important micronutrients such as SiO_2_ (43–90%), Al_2_O_3_ (up to 11%), Fe_2_O_3_ (up to 8%), CaO (up to 22%), and other compounds [36] as well as higher amounts of cellulose (45–50%), hemicellulose (27–29%), and lignin (20–23%) [37]. SBA, as a new low-cost soil amendment, is a micronutrient-rich material and can supply potential opportunities to augment soil organic matter and nutrient content and improve the soil physical, chemical, and biological characteristics, which positively reflect in sustaining crop growth and production as well as help with resilience to abiotic stresses [38].

Based on the justifications above-mentioned, the coupled application of SBA and WD may perform better than sole amendments; however, this co-application for soil remediation has not yet been reported. Our key hypothesis is that the co-application of SBA and WD would modulate the salinity and drought impacts, which could be positively reflected by improvements in the soil nutrient availability and a reduction in the exchangeable sodium percentage, which enhances growth and canola productivity.

## 2. Results

### 2.1. K and Na Ions

#### 2.1.1. Exchangeable Sodium Percentage in the Soil

**Figure 1:**
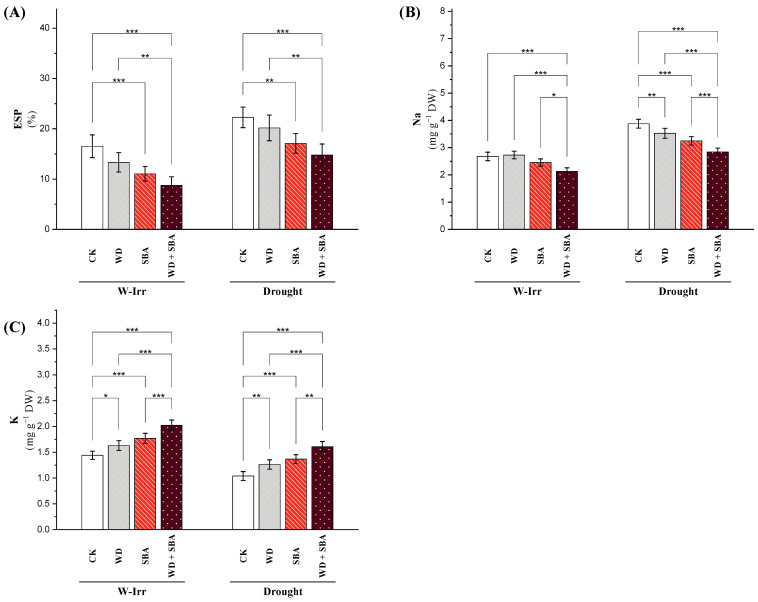
Impact of wood distillate (WD) and sugarcane bagasse ash (SBA) on the exchangeable sodium percentage (ESP) (**A**), and leaf Na and K concentrations (**B**,**C**) in canola plants facing drought stress in salt-affected soil throughout the 2022 and 2023 growing seasons. The data are expressed as the means ± standard deviation, with significance denoted by *, **, and *** for *p*-values less than 0.05, 0.01, and 0.001, respectively. CK = control.

The exchangeable sodium percentage (ESP) value under well-irrigation was 17% for the control group (Figure 1A), whereas a reduction by 18%, 34%, and 48% in the ESP below the control were recorded for treatments involving WD, SBA, and their combination (WD + SBA). On the other hand, drought stress triggered an increase in ESP in all treatments; the control group showed the largest value (23%), indicating a 33% increase in ESP as a result of drought stress. The application of WD, SBA, and their combination reduced the values of ESP compared to the control by 11%, 24%, and 31%, respectively, despite the high ESP during drought, indicating a moderating impact even under stressful conditions (Figure 1A).

#### 2.1.2. Concentration of Na and K Ions

Different treatments and irrigation conditions had a substantial impact on the amount of Na in the canola leaves; for all treatments, there was less Na content under well-irrigated (W-Irr) conditions than under drought conditions (Figure 1B). When drought conditions were controlled by W-Irr, the Na rose from 2.63 to 3.86, showing a 46.7% increase as a result of drought stress. The use of SBA under W-Irr resulted in a 9% decrease in Na concentration. The greatest reduction of 20% in Na content was achieved by the combined use of WD and SBA under W-Irr. In comparison to the control under the same conditions, WD, SBA, and their combined application decreased the Na content by 9%, 16%, and 27%, respectively, under drought conditions (Figure 1B).

In comparison to the drought conditions, canola leaves in the W-Irr conditions consistently had greater K levels across all treatments (Figure 1C). For example, in the control group, the K content decreased by 29% as a result of drought stress from 1.42 under W-Irr to 1.02 during drought. While SBA elevated the K content under the same conditions by 24% to 1.77, the application of WD under W-Irr increased the K content by 13%, from 1.42 to 1.61. The treatment of WD + SBA increased the K content by 40% under W-Irr, which was the most notable rise. WD, SBA, and their combination increased the K content in spite of the drought circumstances by 20%, 32%, and 55%, respectively, above the control (Figure 1C).

### 2.2. Physiological Attributes

#### 2.2.1. Chlorophyll Content and Water Relations

**Figure 2:**
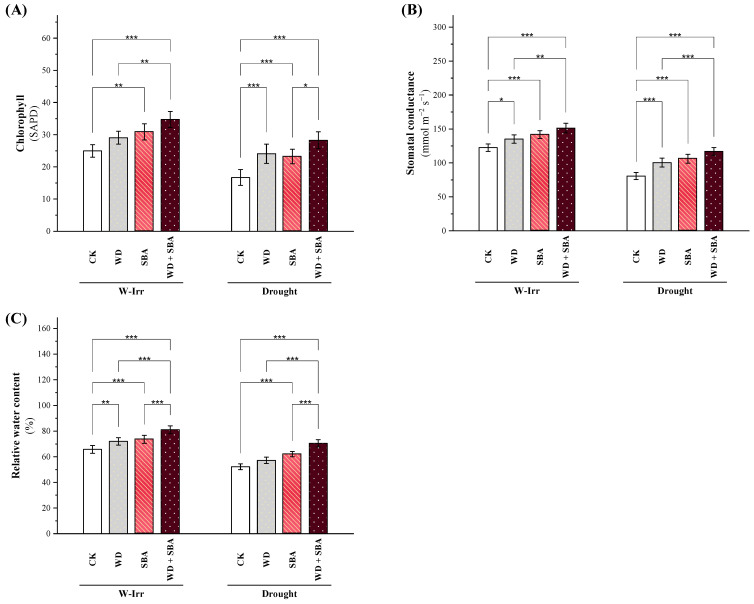
Impact of wood distillate (WD) and sugarcane bagasse ash (SBA) on chlorophyll (**A**), stomatal conductance (**B**), and relative water content (**C**) in the leaves of canola plants facing drought stress in salt-affected soil throughout the 2022 and 2023 growing seasons. The data are expressed as the means ± standard deviation, with significance denoted by *, **, and *** for *p*-values less than 0.05, 0.01, and 0.001, respectively. CK = control.

Based on the SPAD measurements, the amount of chlorophyll in the canola leaves was found to be considerably impacted by the various treatments and irrigation setups. Within each treatment, the W-Irr treatment consistently outperformed the drought conditions. The SPAD values of the control group decreased by 35% as a result of drought stress, from 24.30 under W-Irr to 15.90 under drought conditions (Figure 2A).

The application of WD under W-Irr conditions increased the SPAD values by 19%, to reach 28.92 from 24.30. In a similar trend, the use of SBA resulted in increased values of 30.03, which was 24%. However, the WD + SBA under the W-Irr condition showed the highest increase in SPAD values by 39%, making up 33.76. Applying WD, SBA, and their combination significantly enhanced the SPAD values with a respective increase of 46%, 40%, and 72% compared to that in the control under similar conditions. Remarkably, the increase by the combined application under drought conditions was a substantial 13% above those of the control under W-Irr conditions (Figure 2A).

The stomatal conductance (gs) of canola leaves was significantly affected by different treatment and irrigation conditions, with higher gs values recorded under the W-Irr conditions compared to the drought conditions in all treatments (Figure 2B). For the control, the gs was 120.75 under W-Irr, while it was significantly reduced to 79.15 when drought was applied, representing a reduction of 34% due to drought stress. The gs values changed from 120.75 when WD was applied under W-Irr conditions to 134.05 and rose by 17% in the case of SBA application to reach 141.01. The highest increase of gs, 24% of the values, was in the case with the combined application of WD and SBA under W-Irr conditions to 149.43. The gs values of WD, SBA, and the combination were, under the same situation of drought stress, increased compared to the control. Values of gs increased by 25%, 32%, and very significantly to 47% in the WD treatment, SBA treatment, and the combined application, respectively (Figure 2B). Compared to the combination under drought with the control under W-Irr conditions, the reduction in gs was limited (3.6%).

Significant variations in the content of relative water content (RWC) in the leaves of canola were observed concerning the treatments and irrigation conditions. For instance, under W-Irr conditions, the RWC was always much higher for all treatments than under drought (Figure 2C). In general, the lowest mean RWC was observed in the control, with 65.66 under W-Irr and 52.28 under drought, denoting a reduction of 20% from drought stress. Considerable improvement in RWC was recorded by applying WD under W-Irr by 9%, increasing from 65.66 to 71.80. Similarly, the application of SBA under W-Irr showed an increase in RWC by 11%, from 65.66 to 73.07. The maximum enhancement in RWC was under the combined application of WD and SBA under W-Irr by 24%, to record a value of 81.26 (Figure 2C). In general, all treatments with WD, SBA, and their combination had an improved RWC under drought conditions compared to the control. The application of WD under drought conditions enhanced the RWC by 8% and SBA by 18%, whereas the combined application enhanced the RWC by 34% compared to the control under the same conditions. The combined treatment under drought conditions compared to the control in W-Irr conditions showed a 7% increase in the RWC, signifying that these treatments were effective in reducing the adverse effects of drought stress on the canola leaf water content (Figure 2C).

#### 2.2.2. Oxidative Stress Indicators

**Figure 3:**
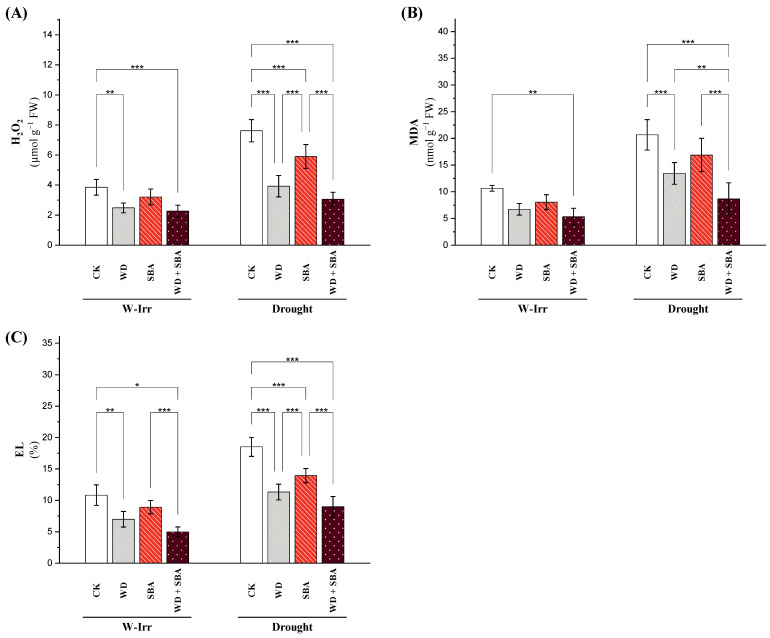
Impact of wood distillate (WD) and sugarcane bagasse ash (SBA) on hydrogen peroxide (**A**), malondialdehyde (**B**), and electrolyte leakage (**C**) in the leaves of canola plants facing drought stress in salt-affected soil throughout the 2022 and 2023 growing seasons. The data are expressed as the means ± standard deviation, with significance denoted by *, **, and *** for *p*-values less than 0.05, 0.01, and 0.001, respectively. CK = control.

The H_2_O_2_ content, a key indicator of oxidative stress in canola leaves, was significantly influenced by the irrigation conditions and different treatments (Figure 3A). In the control group, the H_2_O_2_ content reached 3.66 under W-Irr conditions and surged to 7.45 under drought conditions, with a 104% increase because of drought stress. The application of WD under W-Irr conditions brought down the H_2_O_2_ content by 36%, from 3.66 to 2.33. Similarly, the application of SBA under W-Irr conditions led to a 22% reduction, whereby the H_2_O_2_ levels dropped to 2.85. The combined application of WD and SBA under the W-Irr conditions reduced this parameter by 42%, bringing the H_2_O_2_ level down to 2.10 (Figure 3A). The content of H_2_O_2_ was significantly lowered under drought with the treatment of WD, SBA, and their combination compared with the control under the same conditions. In this regard, applying WD reduced the content of H_2_O_2_ by 51%, that of SBA by 20%, and the combined treatment by 56%. It is essential to underline that, under proper watering conditions, compared to the control, the combined application of treatments under drought was responsible for a decrease of 10% in the content of H_2_O_2_ (Figure 3A).

Malondialdehyde (MDA) content, an indicator of lipid peroxidation and oxidative stress in canola leaves, was highly affected by different treatments and irrigation conditions (Figure 3B). In the control group, the MDA content was 10.48 under W-Irr conditions and increased to 19.57 under drought conditions; that is, it rose by 87%. Applying WD under the W-Irr conditions reduced the MDA content to 37%, from 10.48 to 6.62. The same trend was also observed when SBA was applied with W-Irr conditions, accounting for a 30% reduction in MDA levels, reducing it to 7.34. However, the highest reduction level was observed in the combined application of WD and SBA, with a 42% reduction, reaching the value of 6.03 under W-Irr conditions (Figure 3B). Under drought conditions, the application of WD reduced the MDA content by 38%, SBA by 19%, and a combined application by 50%. Significantly, the MDA content was 7% lower in the combined application than in the control under W-Irr conditions.

There was a regular pattern of electrolyte leakage (EL) of the canola leaves; in all treatments, the EL values under drought were dramatically higher compared to the W-Irr conditions (Figure 3C). Under the W-Irr conditions, the respective EL values for the control were 9.66, which rose to 18.02 under drought conditions and increased by 86%. The application of WD under W-Irr conditions led to a 38% decrease in EL values, dropping from 9.66 to 6.00. Similarly, the application of SBA under W-Irr conditions resulted in a 16% reduction, with the EL values falling to 8.08. The combined application of WD and SBA under W-Irr conditions resulted in the most significant decrease in EL by 54%, bringing the values down to 4.47. The application of WD, SBA, and their combination significantly reduced the EL values even under drought conditions compared with the control under the same conditions. Precisely, WD decreased the EL by 40%, SBA by 27%, and their combined application by 53%. Moreover, combined application decreased the EL by 13% under drought compared to the control under W-Irr conditions (Figure 3C).

#### 2.2.3. Antioxidant Defense System

##### Non-Enzymatic Antioxidants

**Figure 4:**
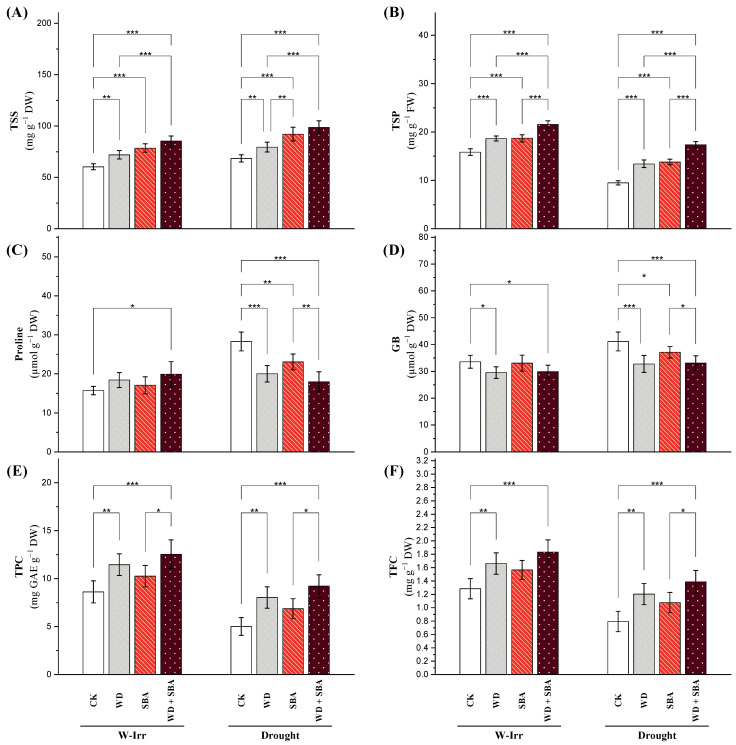
Impact of wood distillate (WD) and sugarcane bagasse ash (SBA) on the total soluble sugars (**A**), total soluble proteins (**B**), proline (**C**), glycine betaine (**D**), total phenolics (**E**), and total flavonoids (**F**) in the leaves of canola plants facing drought stress in salt-affected soil throughout the 2022 and 2023 growing seasons. The data are expressed as the means ± standard deviation, with significance denoted by *, **, and *** for *p*-values less than 0.05, 0.01, and 0.001, respectively. CK = control.

In the control group, the total soluble sugar (TSS) content was 59.7 under W-Irr conditions and increased to 69.51 under drought conditions; this was 16% higher due to drought stress (Figure 4A). Applying WD under W-Irr conditions increased the content of the TSS by 20% to a value of 71.9 from 59.7. Application of SBA under W-Irr conditions increased the TSS by 32%. The highest recorded content of TSS was seen in the combined application of WD and SBA under W-Irr conditions, which was significant at 45%, increasing to 86.7. The increase in the TSS content when the WD, SBA treatments, and their combination were applied under drought conditions was significant compared to the control under the same condition. Significantly, the single application of WD or SBA increased the TSS by 16% and 34%, respectively, while the combined application increased the TSS content by 43%. Notably, the combined application, compared to the control in W-Irr conditions, increased the TSS content by 67% under drought conditions (Figure 4A).

Figure 4B shows that the total soluble protein (TSP) of canola leaves responded obviously to different treatments and irrigations. In the control, the value of 15.9 decreased under drought conditions to 10.01, resulting in a significant 37% decline with drought stress. Applying WD under W-Irr increased the TSP values by 20% to reach 19.1 from 15.9. Similarly, SBA application in the same situation improved the values by 11%, and the TSP reached 17.6. The treatment that showed the largest increase in TSP was the combined application of WD and SBA under W-Irr conditions, increasing the values by 35%. Meanwhile, under drought conditions, the TSP was also increased by combining WD and SBA compared to the drought-stressed control. The application of WD increased the TSP by 51%, while SBA increased it by 41%, and by 83% in the combined treatment. A significant increase of 15% above the control under W-Irr conditions was reported in the case of TSP by the combined application under drought.

Under W-Irr conditions, proline content was 15.4 in the control group and increased to 28.2 under the drought condition (Figure 4C). Under W-Irr conditions, treatment with WD increased the proline content by 18%, whereas SBA application increased the proline content by 10%. Applying both WD and SBA in combination under well-irrigated conditions obtained the highest significant increase in proline content by 25% (Figure 4C). All treatments applied under the drought condition showed decreases in proline content compared to the control. The treatments of WD, SBA, and WD + SBA decreased the proline content by 30%, 19%, and 37%, respectively. Under drought conditions compared to the control, the proline content decreased with different applications, but the WD + SBA treatment showed an increase of 15% over the control under W-Irr conditions (Figure 4C).

The glycine betaine (GB) content of canola leaves showed quite some variation under different treatments and irrigation conditions. For the control group, it was 32.2 under W-Irr conditions, but it rose to 39.5 under drought conditions (Figure 4D). Applying WD, SBA, and their combination under W-Irr conditions resulted in a 12%, 2%, and 10% insignificant reduction in the GB content, respectively. The GB content decreased even under drought stress in the case of WD, SBA, and their combination compared to the control receiving drought stress alone, which reduced the GB content by 20%, 9%, and 19%, respectively. However, the WD + SBA treatment under drought conditions led to a very slight decrease of 0.9% in the GB content (Figure 4D).

The total phenolic (TPC) content in the canola leaves showed remarkable variations in response to different treatments and irrigation conditions, as presented in Figure 4E. In the control group, the TPC content was 9.46 under W-Irr conditions and dropped to 5.72 under drought conditions, marking a remarkable decrease of 40% caused by drought stress. The WD, SBA, and WD + SBA applications under W-Irr conditions increased the TPC content by 30%, 17%, and 42%, respectively, whereas, under drought conditions, the WD, SBA, and WD + SBA treatments enhanced the TPC compared to the control by 55%, 34%, and 77%, respectively. Meanwhile, compared to the W-Irr conditions, the WD + SBA treatment under drought conditions enhanced the TPC content by only 7% (Figure 4E).

The drought stress-induced total flavonoid content (TFC) of canola plants was markedly depressed compared to the W-Irr plants. Plants grown under drought-stress conditions had a 41% lower TFC than their counterparts grown under W-Irr conditions (Figure 4F). This downfall indicates the negative influence of water scarcity on the synthesis of flavonoids in the canola plant. Conversely, under optimum irrigation conditions (80% field capacity), the treatments of WD, SBA, and WD + SBA increased the TFC by 32%, 25%, and 48%, respectively. However, under drought conditions, although the absolute TFC values were brought down compared to the W-Irr conditions, the treatments significantly improved the TFC compared to the drought control. Application of WD, SBA, and WD + SBA increased the TFC by 58%, 40%, and 83%, respectively.

##### Enzymatic Antioxidants

**Figure 5:**
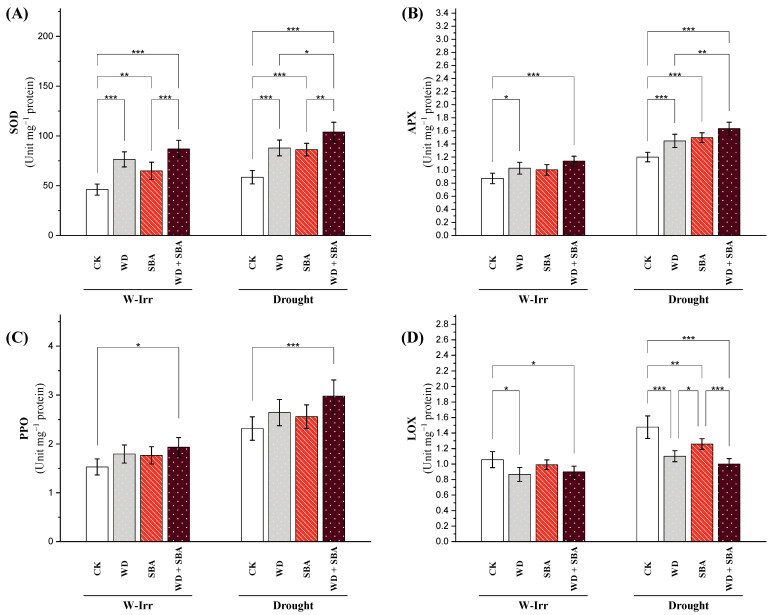
Impact of wood distillate (WD) and sugarcane bagasse ash (SBA) on activity of superoxide dismutase (**A**), ascorbate peroxidase (**B**), polyphenol oxidase (**C**), and lipoxygenase (**D**) in the leaves of canola plants facing drought stress in salt-affected soil throughout the 2022 and 2023 growing seasons. The data are expressed as the means ± standard deviation, with significance denoted by *, **, and *** for *p*-values less than 0.05, 0.01, and 0.001, respectively. CK = control.

Overall, drought stress enabled a significant increase in superoxide dismutase (SOD) activity compared to the W-Irr conditions (Figure 5A). Under W-Irr conditions, all treatments significantly increased SOD activity in comparison to the control. The WD treatment enhanced SOD activity by 69%, while SBA treatment induced SOD activity by 40%. The WD + SBA treatment resulted in a maximum increase of 94% in activity of the SOD. These responses project a strong potential of WD and SBA in strengthening the antioxidant defense system of canola plants under non-stress water supply conditions. Under drought stress, all treatments maintained their significant effectiveness on SOD activity. Application of WD, SBA, and WD + SBA increased SOD activity by 51%, 48%, and 81%, respectively. The WD + SBA treatment under drought conditions was particularly remarkable at elevating the SOD activity up to 137% above that measured in the W-Irr control plants, showing a powerful synergistic mechanism of action in the accumulation of antioxidative defenses under drought stress (Figure 5A).

Ascorbate peroxidase (APX) is a critical enzyme in plants for detoxifying hydrogen peroxide and thereby reducing oxidative stress. APX activity is significantly increased by 38% under drought stress, suggesting enhanced activity of the plant to alleviate the effect of oxidative stress under water deficiency (Figure 5B). Treatments of WD, SBA, and WD + SBA resulted in an increase in APX activity by 18%, 16%, and 30%, respectively, under well-irrigated conditions (Figure 5B). A further increase in APX activity was noted under drought in these treatments. The APX activity increased by 21%, 25%, and 38% as a result of the application of WD, SBA, and WD + SBA, respectively. The WD + SBA treatment resulted in a dramatic increase in APX activity by 91% compared to the control under W-Irr conditions.

Polyphenol oxidase (PPO) is one of the prominent enzymes in the biosystem of plants, having catalytic properties for the oxidation of phenolic substrates. In this way, it contributes to plant defense mechanisms and stress responses. Under drought conditions, the PPO activity increased steeply, with untreated control plants recording a 52% rise in the PPO level compared to the W-Irr conditions (Figure 5C). Furthermore, under W-Irr conditions, the PPO activity was enhanced by the individual application of WD and SBA by 18% and 16%, respectively, over the untreated control. The maximum enhancement in the PPO activity of 28% corresponded to the WD + SBA treatment. Moreover, under drought stress, a 30% increase in the PPO activity corresponded to the WD + SBA treatment compared to the control (Figure 5C).

Lipoxygenase (LOX) is a significant enzyme in lipid peroxidation and an index of oxidative stress and cellular damage in plant tissues. There was a significant increase of 40% in LOX activity concerning the untreated control when the canola plants were exposed to drought stress, which showed that the oxidative stress increased lipid peroxidation due to water scarcity (Figure 5D). Under drought conditions, the decreases in LOX activity were 26%, 15%, and 32%, which corresponded to the treatments of WD, SBA, and WD + SBA, respectively. These findings confirm the effectiveness of such treatments in reducing lipid peroxidation and alleviating oxidative stress under the water deficit. In contrast, under drought conditions, the combined application of WD and SBA led to quite a lower significant increase in LOX activity by 5% compared to the control under W-Irr conditions, thereby reaffirming its potential to keep LOX activity at low levels even under the action of drought-induced oxidative stress (Figure 5D).

### 2.3. Plant Height, Number of Branches and Siliques

**Figure 6:**
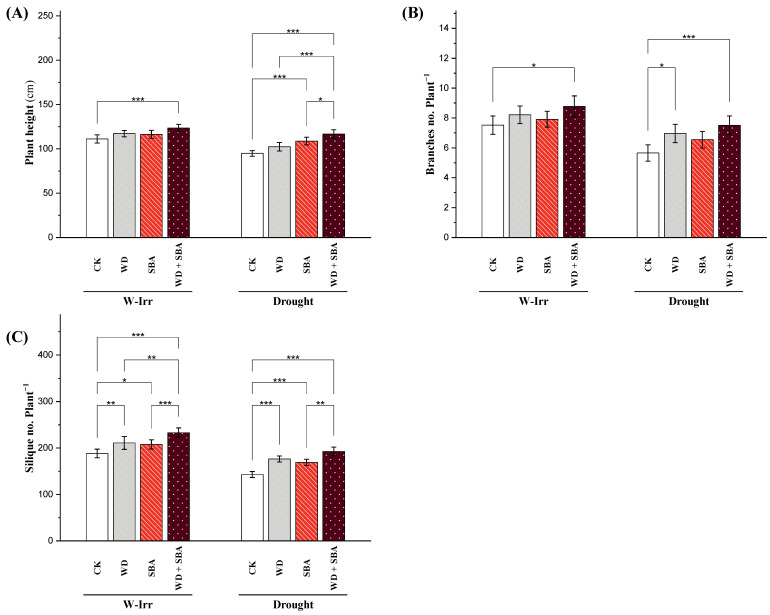
Impact of wood distillate (WD) and sugarcane bagasse ash (SBA) on the plant height (**A**), branches no. plant^−1^ (**B**), and silique no. plant^−1^ (**C**) of canola plants facing drought stress in salt-affected soil throughout the 2022 and 2023 growing seasons. The data are expressed as the means ± standard deviation, with significance denoted by *, **, and *** for *p*-values less than 0.05, 0.01, and 0.001, respectively. CK = control.

Drought stress significantly reduced the plant height in canola to 95.12 cm from 111.42 cm compared to the control treatment (Figure 6A). This reduction reflects the depressing effect of drought stress on the vertical growth of canola plants. Under W-Irr conditions, the treatments resulted in significant increases in plant height compared to the control. A 5% increase in plant height was noticed after the application of WD and SBA. The WD + SBA treatment displayed the highest increase (11%) in plant height. Treatments under drought conditions also significantly enhanced the plant height by 8% with WD application, 15% with SBA application, and 23% with the application of WD + SBA. These results indicate that, even under drought stress, all treatments for the vertical growth of a canola plant improved over the control, whereas the combined treatment was better. The comparison between the combined treatment under drought conditions and the control under W-Irr conditions showed an increase of 5% in plant height, thus indicating the growth-promoting potential of the combined treatment even under water-stressed conditions (Figure 6A).

The number of branches per plant is a critical parameter for canopy architecture and potential yield in canola cultivation. Drought stress significantly reduced the number of branches per plant, with control plants showing a 26% decrease (Figure 6B). Under W-Irr conditions, all treatments significantly increased the number of branches compared to the control, with WD increasing the branches by 10% (from 7.04 to 7.73), SBA by 6% (up to 7.46), and WD + SBA by 18% (reaching 8.30). Under drought conditions, WD increased the number of branches by 24% (from 5.23 to 6.48), SBA by 17% (to 6.11), and WD + SBA by 35% (resulting in 7.04 branches per plant) (Figure 6B).

Drought stress significantly reduced the number of siliques per plant in canola; in the control plants, from 190.13 to 144.71 (Figure 6C). This highlights the severe adverse impacts of drought on canola reproductive development. Under W-Irr conditions, treatments showed a significant difference in the number of siliques per plant compared to the control. Similarly, WD resulted in a 12% increase in silique number per plant, while SBA increased it by 10%. The highest increase (23%) in silique per plant was recorded with the WD + SBA treatment (Figure 6C). Application of WD, SBA, and WD + SBA under water stress increased the number of siliques per plant by 23%, 18%, and 34%, respectively. When the WD + SBA treatment was compared with the control under W-Irr conditions, there was an increase in the number of siliques per plant of 2.3%, which suggests a potential effect as a reproductive promoter, even under limiting water availability (Figure 6C).

### 2.4. Yield Attributes

**Figure 7:**
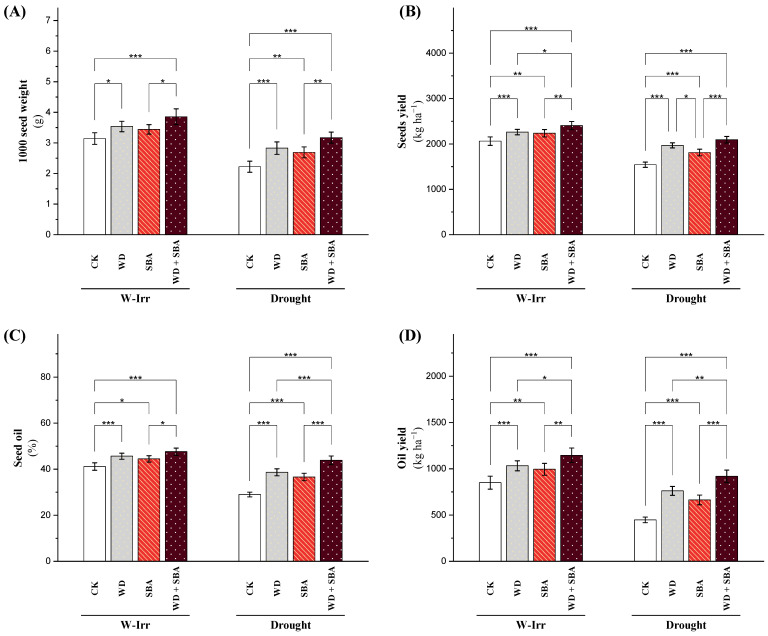
Impact of wood distillate (WD) and sugarcane bagasse ash (SBA) on the 1000 seed weight (**A**), seed yield (**B**), oil percentage (**C**), and oil yield (**D**) of canola plants facing drought stress in salt-affected soil throughout the 2022 and 2023 growing seasons. The data are expressed as the means ± standard deviation, with significance denoted by *, **, and *** for *p*-values less than 0.05, 0.01, and 0.001, respectively. CK = control.

The WD and SBA treatments resulted in a significant alteration in the weight of 1000 seeds, which is an essential parameter for seed quality evaluation and canola cultivation yield potential under W-Irr and drought conditions. The 1000 seed weight decreased by 29% from 3.26 to 2.32 g in the control plants under drought stress, which was a clear indication of the adverse impact of drought on seed development and quality (Figure 7A). The WD treatment significantly increased the 1000 seed weight by 12% (from 3.26 to 3.65 g), SBA by 9% (to 3.55 g), and WD + SBA by 22% (to 3.97 g). It was found that under the effect of drought stress, WD increased the 1000 seed weight by 27% (from 2.32 to 2.94 g), SBA by 20% (to 2.79 g), and WD + SBA by 41% (to 3.27 g). It is also interesting to note that the WD + SBA treatment, even under drought conditions, ameliorated the 1000 seed weight to a level at par with the control under W-Irr conditions (Figure 7A).

Seed yield serves as a good indicator in the estimation of net productivity and economics for canola cultivation. Drought stress caused a significant reduction in the seed yield, as the control plants fell from 2090 to 1568 kg ha^−1^, a decrease of 25% (Figure 7B). Seed yield increased under W-Irr conditions relative to the control in all treatments. The WD, SBA, and WD + SBA treatments increased the seed yield by 9%, 8%, and 16%, respectively. Furthermore, under drought conditions, it was observed that the WD increased the seed yield by 27%, SBA by 17%, and WD + SBA by 35.2%. Remarkably, the interaction effects of WD and SBA (WD + SBA) displayed a seed yield similar to that of the W-Irr treatment control, implying that the treatment alleviated drought stress (Figure 7B).

The oil percentage and oil yield are very vital factors in determining the economics of growing canola because they directly affect both the total quantity and quality of the harvest. Under the drought treatment, the oil percentage and oil yield of the control plants significantly decreased by 30% and 47%, respectively (Figure 7C,D), thus implying a robust negative effect of water stress. However, the treatments of WD, SBA, and WD + SBA significantly improved these parameters under the W-Irr and drought conditions. Under W-Irr conditions, the oil percentage rose by 11% (WD), 8% (SBA), and 16% (WD + SBA), while the oil yield increased by 21%, 17%, and 35% respectively. Under drought conditions, this improvement increased markedly: the oil percentage increased by 33% (WD), 26% (SBA), and 51% (WD + SBA), while the oil yield increased by 70%, 48%, and 105%, respectively. On the other hand, the unfavorable impacts of water stress were lessened under the WD + SBA treatment; oil percentage and oil yield were still significantly higher by 7% and 8%, respectively, compared with the W-Irr control (Figure 7C,D). Thus, the integration of these treatments is specifically effective in elevating oil accumulation and the resulting yield, even in the case of suboptimal water conditions.

### 2.5. Relationship between Physiological and Yield Attributes

**Figure 8:**
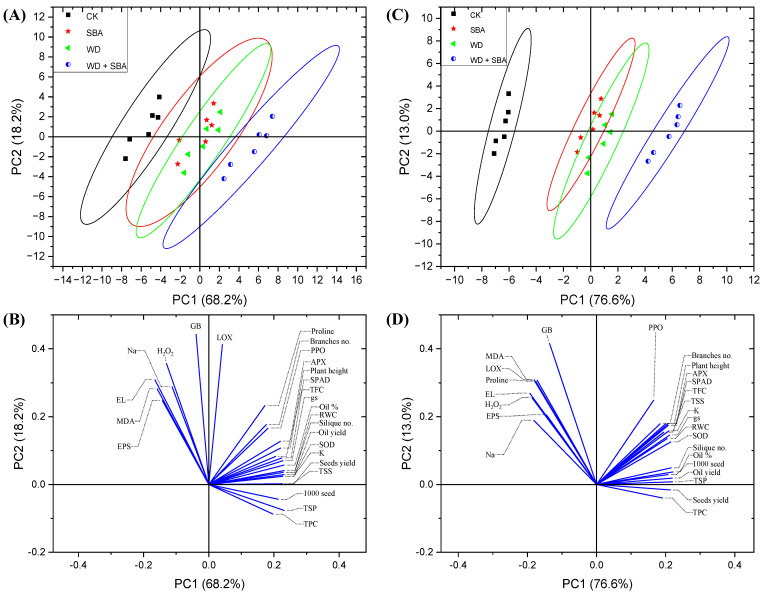
Principal component analysis (PCA) of individual response variables assessed in canola plants subjected to deficit irrigation conditions and the application of wood distillate and sugarcane bagasse. (**A**) [score plot], (**B**) [loading plot] PCA-associated with well-irrigation conditions, (**C**) [score plot], (**D**) [loading plot] PCA-associated with drought conditions.

From the score and loading plots, the principle component analysis (PCA) explained the interactions among different physiological, biochemical, and agronomic parameters of canola under W-Irr and drought conditions (Figure 8). The PCA-associated score plot showed a clear separation between treatments (control and WD + SBA combined-treated) with respect to PC1 (68.2 and 76.6%) and PC2 (18.2 and 13.0%) when the canola plants were irrigated up to 80% FC (W-Irr) and 50% FC (drought) (Figure 8A,C). Under W-Irr conditions, the SBA-treated cluster was clustered in the center of the score plot, which overlapped the margins of the control and the WD + SBA combined-treated clusters, whereas they totally overlapped the WD-treated cluster in the center of the score plot (Figure 8A). Under drought conditions, those WD- and SBA-treated clustered together in the center of the score plot and separately from the other treated clusters (Figure 8C). Furthermore, the PCA-associated loading plot showed that while oxidative stress indicators (MDA, H_2_O_2_, El), EPS, and Na content (Figure 8B) positively correlated with the control treatment under W-Irr conditions (Figure 8A), the antioxidant defense system (SOD, PPO, APX, proline, TSS, TFC, and TPC), plant growth, and yield attributes (plant height, branches number, 1000 seeds weight, seed yield and oil yield) were positively correlated with the WD + SBA treatment (Figure 8A,B). Meanwhile, under drought conditions, the PCA-associated loading plot showed an increase in oxidative stress indicators (MDA, H_2_O_2_, LOX, proline, and El), EPS, and Na content (Figure 8D) that positively correlated with the control treatment under drought conditions (Figure 8C) whereas the antioxidant defense system (SOD, PPO, APX, proline, TSS, TFC and TPC), plant growth, and yield attributes (plant height, branches number, 1000 seeds weight, seed yield and oil yield) were positively correlated with the WD + SBA treatment (Figure 8C,D). Under both irrigation conditions, the PCA-associated loading plot showed that the most correlated variables to seed weight were TSP and TPC (Figure 8B,D). Enzymatic and non-enzymatic antioxidants were positively correlated, suggesting that their increase enhances yield by ameliorating oxidative stress through the ROS scavenging system and osmoprotectant accumulation. Negative correlations were found for MDA and H₂O₂, which are lipid peroxidation and oxidative stress indicators.

**Figure 9:**
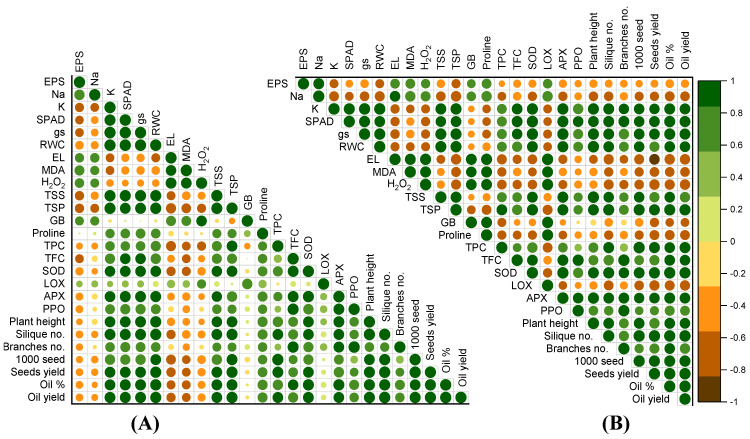
Pearson correlation coefficient of canola physiological, growth, and yield attributes under (**A**) well-irrigation conditions and (**B**) drought conditions. The size of the circle is proportional to the correlation coefficients.

The correlation analysis of canola plants subjected to both W-Irr and drought conditions revealed several significant relationships between various parameters and the seed and oil yields (Figure 9). Under both conditions, the RWC showed a strong positive correlation with the seed yield (r = 0.96464, *p* < 0.001 under W-Irr; r = 0.738, *p* < 0.001 under drought) and oil yield (r = 0.88395, *p* < 0.001 under W-Irr; r = 0.978, *p* < 0.001 under drought). Conversely, the Na content in the leaves was negatively correlated with the seed yield (r = −0.63935, *p* < 0.001 under W-Irr; r = −0.596, *p* < 0.01 under drought) but positively correlated with the oil yield (r = 0.71964, *p* < 0.001 under W-Irr), highlighting contrasting effects depending on the yield type. Additionally, the K content in leaves, chlorophyll levels (SPAD values), and gs showed negative correlations with the seed yield but positive correlations with the oil yield under W-Irr. Under drought conditions, TSS and GB were positively correlated with both yields, while negative correlations were observed with chlorophyll content, gs, EL, and MDA. Interestingly, H_2_O_2_ exhibited very strong positive correlations with both yields under W-Irr conditions but showed negative correlations with oil yield under drought. The analysis underscores the importance of parameters such as RWC, TSS, and SOD in enhancing canola productivity under varying irrigation conditions, while also highlighting the need to manage stress indicators like Na content, chlorophyll levels, and EL to mitigate adverse effects, particularly under drought conditions (Figure 9).

## 3. Discussion

One of the most daunting challenges facing the world is achieving food self-sufficiency for a growing population. The depletion of soil fertility and irrigation water in arid and semi-arid regions around the world is causing anxiety for crop productivity. Therefore, salt-affected soil and water stress are the classical issues that impede the development of plants and metabolic rate, instigating severe reduction in crop production due to soil degradation, which needs smart management for sustainable crop production. The decrease in the soil’s organic matter content and the lack of regular irrigation water for the soil are the main causes of soil salinity. To improve soil health, soil salinity must be treated, and the soil must be compensated for the lack of irrigation water by adding some organic amendments derived from plant origin, which contribute to adding beneficial nutrients to the soil that have been found to be worthwhile.

In this research, it was shown that applying sugarcane bagasse with wood distillate achieved higher results than applying SBA or WD alone. It was observed in our report that plants grown in soil not amended with SBA and foliar spraying with WD that served as the control (untreated plants) suffered from oxidative stress, and it was shown that the contents of oxygen radicals such as H_2_O_2_ were higher under water stress conditions and that the salt-affected soil resulted in a negative impact on the growth and productivity of canola [38]. Meanwhile, the antioxidant activity and related traits were significantly improved, while ROS decreased under water stress conditions and soil salinity when coupled with the application of SBA and WD [29]. The principal technique employed to improve the soil fertility used for these crops is through the application of soil amendments such as SBA, which showed a noticeable improvement in the soil’s physical and chemical properties [39]. The second technique employed to improve plant growth and development under water stress for these crops is through foliar spraying with WD, which showed an increase of antioxidant activities, which help in eliminating ROS and increase the physiological parameters, improving the nutrient uptake through the xylem and phloem to the leaves and seeds; these findings are in conformity with Ma et al. [28]. Applying WD, SBA, and their combination significantly enhanced the SPAD values with a respective increase of 46%, 40%, and 72% compared to the control under similar conditions. Remarkably, the increase by the WD + SBA treatment under drought conditions was a substantial 13% above those of the control under W-Irr conditions (Figure 2A).

Under salt-affected soil and water stress conditions, it was observed that soil amendment with SBA positively impacted on increasing the soil enzyme activities (dehydrogenase, urease, and phosphatase) as well as decreasing the exchangeable sodium (ESP) percentage, which improved the microbial community through osmotic and ionic equilibrium, increased water holding capacity, and the hydration of microbial cells [40]. Additionally, it was found that SBA augmented the plant-available nutrient and cation exchange capacity [34]. The application of SBA positively impacted the ion exchange and insoluble soil physicochemical properties and also caused a significant increase in C and N cycling [41]. The positive role of SBA was attributed to the negatively charged functional groups (including carboxyl, phenolic hydroxyl and carbonyl groups) on the surface of SBA [42]. Our results demonstrate that the improvement in soil enzyme activity by improving the water retention and increasing the soil microorganisms resulting in soil amendment was due to the higher soil-specific surface area of SBA, which decreased the salinity stress under water stress [43]. The application of WD, SBA, and their combination reduced the values of ESP compared to the control by 11%, 24%, and 31%, respectively, despite the high ESP during drought, indicating a moderating impact even under stressful conditions (Figure 1A).

Our study showed that the application of SBA ameliorated water retention and resulted in mitigated soil salt stress in the soil solution, therefore decreasing the osmotic damage, which could indirectly improve the ion balance in the leaves and chlorophyll content (SPAD value) [35]. The analysis of SBA contained high N, P, and K concentrations and other nutrients, which stimulated the soil nutrient concentrations [38]. Increased soil nutrient concentrations positively reflected the physiological attributes in canola plants such as gs, RWC, and decreased proline content [44]. It was obvious that the application of SBA had the potential to increase the antioxidant enzyme activities in the leaves while decreasing the oxidative stress (MDA and H_2_O_2_) under abiotic stress conditions, which is an important gateway to maintaining the assimilation of CO_2_, water, and nutrients. Under drought conditions, the application of WD reduced the MDA content by 38%, SBA by 19%, and WD + SBA by 50%. Significantly, the MDA content was 7% lower in the WD + SBA treatment than in the control under W-Irr conditions.

According to these results, it was found that soil application with SBA could increase osmoprotectants such as TSS, TPC, and TFC as well as improve biochemical traits such as TSP under these harsh conditions [36]. According to these findings, it was attained that the yield and yield components (number of siliques, number of branches, 1000-seed weight and seed yield) as well as oil content increased in canola under water stress in salt-affected soil when SBA was applied.

In addition, foliar application with natural substances such as WD is one approach to stimulate plant resistance to environmental stressors [45]. It was found that WD has organic acids including acetic acid, formic acid, and propionic acid as well as a pH level of 2–4. This makes it suitable for foliar spraying on the plant and can be used as a natural growth stimulator that is safe for the environment [23]. A notable improvement in soil and plant health was observed with the application of WD because aldehydes, ketones, alcohols, esters, and trace metals like potassium, calcium, magnesium, and iron as well as acids and phenols were found in its components [46]. Our results also found that WD under abiotic stress increased the activity of antioxidant enzymes and reduced oxidative stress, leading to an improvement in the physiological state of rapeseeds under water stress in salt-affected soils due to phenolic support involved in the synthesis of WD because of its vital function in inhibiting oxidative damage [26]. In addition, an increase in the vitality of the plant roots, the balance of ions inside the leaves (K/Na), and improved physiological and biochemical processes were found [25]. The greatest reduction of 20% in Na content was achieved by the WD + SBA treatment under W-Irr conditions. In comparison to the control under the same conditions, WD, SBA, and WD + SBA decreased the Na content by 9%, 16%, and 27%, respectively, under drought conditions (Figure 1B).

The impact of the application of WD on physiological processes, SPAD, ion leakage, biochemical processes and osmolytes, and in plant development is all promising. These positive benefits from the application of WD are due to its turn in augmenting the antioxidant enzyme activity, showing that the plants dealt with stress by scavenging damaged ROS [47]. The WD + SBA treatment under drought conditions was particularly remarkable at elevating the SOD activity up to 137% above that measured in the W-Irr control plants, showing a powerful synergistic mechanism of action in the accumulation of antioxidative defenses under drought stress (Figure 5A).

Under water stress, cellular molecules and intrinsic membrane properties were damaged due to the over-accumulation of ROS such as H_2_O_2_ and lipid peroxidation [45]. However, the application of WD caused an increase in antioxidant enzyme activities, which helped in H_2_O_2_ detoxification and converted it into water and oxygen with the help of the antioxidant defense system [48]. It is essential to underline that, under proper watering conditions, compared to the control, the WD + SBA treatment under drought was responsible for a decrease of 10% in the content of H_2_O_2_ (Figure 3A).

Under water stress, it was found that proline production was high, which means that there was an imbalance of osmotic pressure; however, plants treated with WD experienced lower proline accumulation. This result was in conformity with prior reports. Under water stress treatment, a decrease in chlorophyll content, physiological processes (RWC and gs) and increase leave Na/K was found, which decreased the osmoprotectants (TPC and TFC) as Na affected the water and nutrient uptake, while treating plants subjected to water stress with WD recovered their health status in canola plants due to raised K^+^ ions [49]. Values of gs increased by 25%, 32%, and very significantly to 47% in the WD, SBA, and WD + SBA treatments, respectively (Figure 2B). Compared to the WD + SBA treatment under drought with the control under W-Irr conditions, the reduction in gs was limited (3.6%).

It was found that organic acids and other alcohol derivatives that are found in WD encourage nutrient and water transportation from the roots to leaves [31]. WD’s butenolidine concentration and acidity increased the vegetative growth, augmented leaf shading on the soil, and therefore triggered incremental soil moisture content that resulted in improved tolerant to salt stress [50]. In addition, WD could enhance the soil acidity and CEC, which aided in enhancing nutrient availability [51]. Under water stress, all yield related traits reduced. However, it was observed that the number of siliques, number of branches, 1000-seed weight, seed yield, and oil quality were significantly augmented with WD application under water stress in salt-affected soil [52].

Our results showed that when plants are subjected to salt stress and water stress together, WD coupled with SBA induced higher leaching of soluble salts that resulted in a higher improved soil structure and moisture content, which ultimately decreased the effects of salt and water stress on the plant and also caused a higher increase in plant growth, productivity, and quality compared to the sole application of either WD or SBA.

## 4. Materials and Methods

### 4.1. Experimental Layout

Two field experiments were performed to evaluate the co-application of soil amendment by waste SBA and foliar spraying by WD on canola plants grown under water stress conditions in salt-affected soil during the 2021/2022 and 2022/2023 winter seasons at the Elamaar village in the region of Sidi Salem (31° 07 N, 30° 57 E), Kafr El-Sheik Governorate, Egypt. The experiment was laid out as a split-plot design with three replicates where irrigation levels were in the main plots, while the amendment treatments (control, WD, SBA, and WD + SBA) were used as subfactors. Canola seeds (*Brassica napus* L., cv. Bactol) were attained by the Oil Research Department, Sakha, Kafr El-Sheikh, Egypt, with an application rate of 7 kg seeds ha^−1^, which were cultivated on 15 November 2021 and repeated on 20 November 2022. The experimental areas were divided into plots 4 m long × 3 m wide. Three seeds were planted per hill in each experimental plot at a depth of 5 cm with a 25 cm plant spacing and 30 cm between ridges, which were thinned to one seedling per hill after full germination (120,000 plants density ha^−1^) and a 1 m alley between treatments. The mineral fertilizers of NPK were added to all experimental units. Calcium superphosphate fertilizer was mixed into the soil at a rate of 36 kg P_2_O_5_ ha^−1^ with the second plowing. Then, nitrogen fertilization (as ammonium nitrate, 33.5% N), applied at a rate of 350 kg N ha^−1^, was divided into two equal doses and added before the first and second irrigations. Potassium fertilization was added at a rate of 58 kg ha^−1^ (K_2_O) before the second irrigation. Prior to sowing, the experimental plot was plowed and disked to attain a level seedbed. Weed control was carried out by hand. No pests or diseases appeared during both seasons. The irrigation treatments were well-irrigation (W-Irr: 80% field capacity) and deficit-irrigation to express the drought stress (50% field capacity). The soil water content was controlled through tensiometers. The average temperature, rainfall, and relative humidity for the cultivated zone were measured during the 2021–2022 and 2022–2023 winter seasons, as indicated in Table 1.

The soil was attained from the experimental farm at a 0–30 cm depth from Elamaar village in the region of Sidi Salem; then, the soil was air-dried and sieved through a 2 mm steel mesh for the analysis of the physicochemical characteristics, as displayed in Table 2.

#### 4.1.1. SBA Characterization

The bagasse feedstock was obtained from the sugarcane factory in Upper Egypt (the main area of sugarcane production in Egypt), air-dried at room temperature (20–25 °C), and finally ground to 2 mm. The raw bagasse material was thermally combusted slowly at 400 °C in a muffle furnace for 2 h under a limited oxygen environment. Prepared SBA was stored in a container and used as the soil amendment. SBA was applied to the soil pre-planting at a rate of 10 tons ha^−1^. SBA was plowed to a depth of 3–5 cm in the soil. The basic physicochemical properties of the raw bagasse were analyzed based on Arshad et al. [53] as follows: pH (1:20) (suspension) 9.2, electrical conductivity (1:20) extract 1.02 dS m^−1^, capacity exchange capacity 4.01 cmolc kg^−1^, specific surface area 10.2 m^2^ g^−1^, carbon 527 g kg^−1^, nitrogen 4.9 g kg^−1^, sulfur 0.40 g kg^−1^, C:N ratio 127, iron 680 mg kg^−1^, zinc 88.1 mg kg^−1^, potassium 5.95 g kg^−1^, phosphorus 186.02 mg kg^−1^, sodium 1.16 g kg^−1^, calcium 27.7 g kg^−1^, magnesium 8.06 g kg^−1^, and organic matter 25.9%.

#### 4.1.2. Wood Distillate Characterization

The WD extract was obtained from the Department of Agricultural Microbiology, Soils, Water, and Environment Research Institute (SWERI), Agricultural Research Center (ARC), Egypt. A 200-fold diluted WD (1%) was used on the surface of the canola leaves, with Tween 80 at 0.1% used as a surfactant, and sprayed three times (30, 50, and 70 days after sowing) using a small handheld sprayer. The main component of WD was measured by atomic absorption spectrophotometry (AAS Model GBC 932 Plus) and gas chromatography-mass spectrometry (GCMS-QP2010 Shimadzu, Kyoto, Japan). The selected properties of WD after chemical analysis were as follows: pH (2.95), TDS (3.22 S cm^−1^), sulfur (1.13%), K (1765 ppm), Ca (386 ppm), Mg (45 ppm), Fe (1.75 ppm), P (28 ppm), Zn (2.83 ppm), Cu (1.48 ppm), water (68%), organic matter (27.9%). The selected properties of WD after organic matter profile analysis were as follows: phenols and derivatives (36.45%), alkane compounds (3.28%), furan derivatives (1.39%), acetic acid (9.87%), organic acid (31.30%), aldehydes (1.86%), formic acid (0.318%), alcohol (10.24%), ketones (9.45%), and esters (5.71%).

### 4.2. Measurements

#### 4.2.1. Exchangeable Sodium Percentage (ESP)

Ten soil samples were randomly chosen from each experimental plot at harvest by an auger and oven-dried to analyze Na, Ca, and Mg in a water-saturated soil extract (meq L^–1^) by an atomic absorption spectrophotometer (AAS, PERKIN ELMER 3300) to calculate the soil Na adsorption ratio (SAR), followed by calculating the ESP as the following empirical equation, according to Arshad et al. [53]:

$$ESP=1.95+1.03\times SAR\;(R^{2}=0.92)$$

where the SAR (sodium adsorption ratio) was calculated using Equation (1)

(1) $$SAR=\left[Na^{+}\right]/\sqrt{\frac{\left(\left[C{a^{2}}^{+}\right]+\left[M{g^{2}}^{+}\right]\right)}{2}}$$

where Na^+^, Ca^2+^, and Mg^2+^ are expressed in meq L^−1^.

#### 4.2.2. Leaf Na^+^ and K^+^ Determination

At 85 days after sowing, ten leaves were chosen randomly from each experimental unit to assess the Na^+^ and K^+^ concentrations (mg g^−1^ DW) with ultra-pure water. The volume of the sample was applied to 50 mL in a volumetric flask. Based on the Temminghoff and Houba method [54], the Na^+^ and K^+^ contents were estimated by AAS (Perkin Elmer 3300, Waltham, MA, USA) with a detection limit of 100 ppb.

#### 4.2.3. Chlorophyll Content (SPAD Reading)

At 85 days after planting, ten plants in each experimental plot were randomly selected to determine the SPAD value from the fully expanded uppermost leaves. For the determination of chlorophyll, a meter (model SPAD-502, Minolta Sensing Ltd., Konica Minolta Sensing, Inc., Headquarters: Sakai, Osaka, Japan) was used as assessed by Ling et al. [55].

#### 4.2.4. Determination of Relative Water Content and Stomatal Conductance

At 85 days after planting, ten plants from each experimental plot were randomly selected for the determination of stomata conductance (gs, mmol H_2_O m^−2^ s^−1^). The gs was measured in the youngest fully expanded top leaf between 9:00 and 11:00 am using LI-6400 portable gas exchange systems from Li-COR, Lincoln, NE, USA.

The relative water content, RWC (%), was determined using additional selected samples. Leaf disks of 1 cm² area were used for the experiment. The fresh weight, FW, of those disks was noted. The turgid weight, TW, was determined by leaving the leaf disks in distilled water to soak for five hours. The dry weight, DW, was then obtained by oven drying at 70 °C for 48 h. The RWC was estimated from the following empirical equation by Weatherley [56]:

RWC%=(FW−DW)/(TW−DW)×100

#### 4.2.5. Electrolyte Leakage, Lipid Peroxidation, and H_2_O_2_

Simultaneously, samples were taken to determine the electrolyte leakage (EL, %). Flat disks of leaves with an area of 1 cm² were washed with tap water and moved to distilled water, afterward transferring them to test tubes, each having 10 mL of distilled water. The EL was determined using the following empirical formula, as proposed by Bajji et al. [57]:

EL%=C1/C2×100

where *C*1 is the conductivity of samples maintained in a water bath at 55 °C for 25 min, and *C*2 is that of samples maintained in a water bath at 100 °C for 10 min.

The malondialdehyde (MDA) content (nmol g^−1^ FW) was measured by the method of Du and Bramlage [58] using the thiobarbituric acid test. The peroxidation of lipids was measured using 250 mg of frozen leaves homogenized and ground in liquid nitrogen with hydro-acetone buffer (4:1, *v*/*v*). The medium was mixed with a 20% TCA solution, 0.01% butyl hydroxyl toluene, and heated at 95 °C, followed by centrifugation at 10,000× *g* for 10 min. The absorbance of the supernatant was measured spectrophotometrically at 532 nm and 600 nm in a Shimadzu (Japan) UV-160A spectrophotometer.

The hydrogen peroxide content (H_2_O_2_, µmol g^−1^ FW) in relation to 1 g of the fresh mass of the examined leaves was analyzed in accordance with Velikova et al. [59], with fresh material homogenized in liquid nitrogen and aqueous trichloroacetic acid (0.1% TCA) and then centrifuged for 15 min at 6000× *g*. The level of H_2_O_2_ was analyzed colorimetrically based on the absorbance level of 426 nm using a UV-160A spectrophotometer (Shimadzu, Kyoto, Japan).

#### 4.2.6. Proline, GB, TSS, TSP, TPC, and TFC

At 85 days after sowing, samples were chosen to determine the proline content (µmol g^−1^ DW) based on Bates et al. [60]. Plant leaves (0.5 g) were ground with 3% sulfuric acid and centrifuged at 12,000× *g* for 5 min. The solution was reacted with ninhydrin reagent, extracted with toluene, and the absorbance was determined at 520 nm using a UV-160A spectrophotometer (Shimadzu, Japan).

##### Glycine Betaine (GB, µmol g^−1^ DW)

At the same time, 0.5 g of the leaves was randomly selected and extracted by shaking with 15 mL distilled water for 24 h at 25 °C according to the method of Grike and Grattan [61] to quantify the glycine betaine (GB, µmol g^−1^ DW) content. The filtered extract was diluted 1:1 with 1 M sulfuric acid, followed by chilling and reacting with a KI-I_2_ reagent (1 mL) for 1 h in ice water at 4 °C. The absorbance was then measured at 365 nm following centrifugation for 15 min at 0 °C and 10,000× *g*.

Samples of the leaves (0.5 g) were randomly selected and extracted with 80% hot ethanol following Sadasivam and Manickam [62] to measure the total soluble sugar (TSS, mg g^−1^ DW). An aliquot of 0.1 mL was mixed with 1 mL anthrone reagent (200 mg anthrone was dissolved in 100 mL of 95% sulfuric acid and incubated at 100 °C). The absorbance was measured at 625 nm.

Total soluble protein (TSP, mg g^−1^ FW) was determined using the Bradford method [63]. The leaf samples were homogenized, and TSP was determined by the reaction with Coomassie Brilliant Blue dye, read at an absorbance of 595 nm.

The determination of total phenolic content (TPC mg g^−1^ DW) was based on Baydar et al. [64]. A total of 2 g of the dried root and shoot samples was crushed in 90% methanol in a shaker for one hour. The extract was centrifuged, then diluted and mixed with Folin–Ciocalteu reagent in the ratio 4:1. Afterward, 10% Na_2_CO_3_ was added and kept in the dark for one hour; the absorbance was later read at 760 nm using a spectrophotometer.

The total flavonoid content (TFC mg g^−1^ DW) was estimated by the colorimetric aluminum chloride (AlCl_3_) method based on the procedure described by Chang et al. [65]. The dried plant material (0.1 g) was mixed with 0.5 mL of 1:10 methanol, 1.5 mL of methanol, 0.1 mL of potassium acetate, 0.1 mL of 10% AlCl_3_, and 2.8 mL of water. The mixture was left to stand at room temperature for 30 min, and finally, the absorbance was recorded at 415 nm using a spectrophotometer.

#### 4.2.7. SOD, APX, PPO, and LOX

Samples of canola leaves (1 g each) were ground and homogenized in 5 mL of cold phosphate buffer (50 mM phosphate buffer pH 7.0 containing 0.5 mM EDTA, and 1% polyvinylpolypirrolidone) that was used as an enzyme extract. Afterward, the homogenized solutions were centrifuged for 25 min at 10,000× *g* at 4 °C to use the supernatant as an enzyme extract [66,67].

##### Superoxide Dismutase (SOD Units mg^−1^ Protein)

In the present study, the activity of superoxide dismutase (SOD units mg^−1^ protein) was measured by a photochemical nitro-blue tetrazolium, NBT, assay at 560 nm following the Beauchamp and Fridovich method [68].

The activity of ascorbate peroxidase: EC 1.11.1.11 (APX units mg^−1^ protein) was measured by the method of Jebara et al. [69], with some modifications. In brief, the reaction mixture contained 50 mM of potassium phosphate buffer at a pH of 7.0, 0.5 mM ascorbic acid, 0.1 mM H_2_O_2_, and 30 μL of enzyme extract in a total volume of 3 mL. The concentration of oxidized ascorbate was determined by the decrease in absorbance at 290 nm using an extinction coefficient of 2.8 mM^−1^ cm^−1^.

The activity of polyphenol oxidase: EC 1.10.3.1 (PPO Units mg^−1^ protein) was assayed according to the technique described by Raymond et al. [70]. The reaction mixture consisted of 2.7 mL of 200 mM sodium phosphate buffer, pH 6.8, 0.2 mL of 20 mM pyrogallol, and 100 μL enzyme extract. The increase in absorbance was recorded at 430 nm.

The activity of lipoxygenase: EC 1.13.11.12 (LOX Units mg^−1^ protein) was measured according to Grossman and Zakut [71]. Pure linoleic acid (10 µL) was dispersed in 25 mL of 0.1 M sodium tetraborate containing 0.1% Tween 20. Then, 0.1 mL of the substrate was vortexed thoroughly with 2.8 mL of 0.1 M phosphate buffer, pH 5, in a spectrophotometer cuvette. The reaction was initiated by the addition of 0.1 mL of the enzyme extract, and an increase in the absorbance at 234 nm was measured for activity.

#### 4.2.8. Attributes of Vegetative Growth and Oil Yield

At harvesting time, ten plants were collected from each experimental unit to assess the yield characteristics (i.e., plant height, number of siliques plant^−1^, and number of branches plant^−1^), while plants of whole plots from each treatment were collected to assess the seed yield. The harvested seeds were stored after the seeds were manually threshed and left to dry. The weight of 1000 seeds (g) and the seed yield adjusted to kg ha^−1^ were estimated at a moisture basis of 10%. To assess the oil percentage (% dry matter) in the seed samples, a Soxhlet extractor method was used [72]. The oil yield (kg ha^−1^) was then computed using multiplying the seed yield (kg ha^−1^) by the seed oil content.

### 4.3. Statistical Analysis

An elaborate statistical analysis was conducted to evaluate the influence of four treatments, SBA, WD, SBA+WD and CK, under two irrigation conditions, 80% FC and 50% FC, in a split-plot design over two years, on the various physiological and biochemical parameters in canola. Descriptive statistics, in the form of means and standard deviations for all measured parameters, were computed. ANOVA was carried out, followed by post hoc comparisons using Tukey’s HSD test, to show significant differences. The relationships between each pair of parameters under study were estimated for well-irrigated and drought conditions by calculating the coefficients of the Pearson correlation. Principal component analysis was applied to reduce the dimensionality and determine the principal components explaining a dataset variance. Statistical analyses were conducted in CoStat (package 6.45, CoHort, Monterey, CA, USA) and R version 4.4.1.

## 5. Conclusions

The combined application of SBA and WD holds great potential to improve the growth and performance of canola under drought conditions in salt-affected soils. Based on our two-year field experiment involving a split plot design, there were positive effects of individual and combined treatments of SBA and WD on the physiological responses of canola and its yield. Specifically, WD combined with SBA conferred the maximum benefits by improving the soil properties, water retention, and nutrient availability, thus alleviating the negative impacts of drought stress. Under drought stress, the combination postured was clearly better than the control and the other single factors in maintaining higher photosynthetic rates, better water-use efficiency, and enhanced biomass production. These findings show that the WD + SBA treatment has huge potential as a sustainable agronomic practice to improve the resilience and productivity in canola under challenging growth conditions—a lead-in into more resilient agricultural systems under rising climate variability.

## Figures and Tables

**Table 1:** The average temperature, rainfall, and relative humidity during the 2021–2022 and 2022–2023 winter seasons.

Year Month	2021/2022				2022/2023			
	Temperature (°C)		Rainfall (mm)	Relative Humidity (%)	Temperature (°C)		Rainfall (mm)	Relative Humidity (%)
	Max	Min			Max	Min		
November	25.52	16.44	1.02	34.56	26.06	16.2	1.48	32.14
December	24.42	14.33	1.15	36.22	24.96	14.3	1.36	33.74
January	23.32	12.76	1.25	37.14	23.86	12.4	1.08	33.24
February	21.74	9.44	2.48	47.36	22.28	11.1	3.86	42.94
March	22.36	10.67	1.48	46.47	22.9	10.7	7.39	43.64
April	23.14	11.69	0.89	45.65	23.68	12.5	1.17	45.34
May	24.36	12.48	0.75	44.25	24.9	16.2	0.54	44.54

**Table 2:** Analysis of the physicochemical characteristics of the experimental site during the 2021/2022 and 2022/2023 seasons.

							Cations (meq L^−1^)				Anions (meq L^−1^)		
	Season	OM (%)	Soil Texture	EC (dS m^−1^)	FC (%)	pH	Na^+^	K^+^	Mg^+2^	Ca^+2^	Cl^−^	HCO_3_^−^	SO_4_^−2^
Soil	2021/2022	1.24	Clayey	8.33	32.56	8.99	18.01	10.96	14.99	16.77	23.79	17.45	19.26
	2022/2023	1.38	Clayey	7.87	33.23	8.67	18.88	12.09	16.46	18.52	24.44	19.9	21.38

OM = organic matter; EC = electric conductivity; FC = field capacity.

## Data Availability

Data are contained within the article.
